# Can usual gait speed be used as a prognostic factor for early palliative care identification in hospitalized older patients? A prospective study on two different wards

**DOI:** 10.1186/s12877-020-01898-w

**Published:** 2020-11-24

**Authors:** Celine Van de Vyver, Anja Velghe, Hilde Baeyens, Jean-Pierre Baeyens, Julien Dekoninck, Nele Van Den Noortgate, Ruth Piers

**Affiliations:** 1 Department of Geriatric Medicine, Ghent University Hospital, C. Heymanslaan 10, 9000 Ghent, Belgium; 2 Department of Internal Medicine and Pediatrics, Ghent University, Ghent, Belgium; 3 Department of Geriatric Medicine, AZ Alma Eeklo, Eeklo, Belgium; 4 Department of Geriatric Medicine, Sint-Andriesziekenhuis, Tielt, Belgium

**Keywords:** Usual gait speed, Palliative care, Advance care planning, One-year mortality

## Abstract

**Background:**

Timely palliative care in frail older persons remains challenging. Scales to identify older patients at risk of functional decline already exist. However, factors to predict short term mortality in older hospitalized patients are scarce.

**Methods:**

In this prospective study, we recruited patients of 75 years and older at the department of cardiology and geriatrics. The usual gait speed measurement closest to discharge was chosen. We used the risk of dying within 1 year as parameter for starting palliative care. ROC curves were used to determine the best cut-off value of usual gait speed to predict one-year mortality. Time to event analyses were assessed by COX regression.

**Results:**

On the acute geriatric ward (*n* = 60), patients were older and more frail (assessed by Katz and iADL) in comparison to patients on the cardiology ward (*n* = 82); one-year mortality was respectively 27 and 15% (*p* = 0.069). AUC on the acute geriatric ward was 0.748 (*p* = 0.006). The best cut-off value was 0.42 m/s with a sensitivity and specificity of 0.857 and 0.643. Slow walkers died earlier than faster walkers (HR 7.456, *p* = 0.011), after correction for age and sex. On the cardiology ward, AUC was 0.560 (*p* = 0.563); no significant association was found between usual gait speed and survival time.

**Conclusions:**

Usual gait speed may be a valuable prognostic factor to identify patients at risk for one-year mortality on the acute geriatric ward but not on the cardiology ward.

**Supplementary Information:**

The online version contains supplementary material available at 10.1186/s12877-020-01898-w.

## Background

The last decades, the rapid ageing of our society brings an increasing prevalence of chronic diseases and frailty, thereby raising the number of patients with higher dependency at the end of life [1]. Of all the persons of 75 years and older, 30–60% have a geriatric risk profile. An acute event occuring in this group of frail older patients often results in a need for hospitalization [2–4]. Most of these patients will be admitted on the acute geriatric ward, known for its focus on comprehensive geriatric assessment, early rehabilitation, early discharge planning, and person-centered care within a multidisciplinary team [5]. However, some of them will also be admitted on non-geriatric wards in which there is a more single-disease approach [5].

Frail patients are at increased risk of adverse outcomes as dependency, falls, institutionalization and also mortality [6, 7]. In this subgroup, the implementation of early palliative care could be beneficial. Palliative care, defined by the WHO, is an approach that improves the quality of life of patients and their families facing problems associated with life-threatening illness, through the prevention and relief of suffering by means of early identification and assessment and treatment of pain and other problems, physical, psychosocial and spiritual [8].

Early integration of palliative care and advance care planning diminishes anxiety, stress, and depressive feelings in patients and families [9–14]. Families have more open dialogues on the subject of end-of-life and more patients die on the place of their preference. On top of that, inappropriate diagnostic and therapeutic procedures are avoided [9].

Although proven to be beneficial, the implementation of palliative care in frail older persons remains suboptimal, probably because in this subgroup, the end-of-life trajectory is often difficult to predict [11]. Prognostic uncertainty therefore causes physicians to be less confident in making the transition from cure to care [13–17].

To better identify frail older patients at higher risk of dying, prognostic factors and tools are important [15, 16]. Some of them, as the Interstitial Lung Disease-GAP model [17] and the Seattle Heart Failure Model [18] are examples of organ specific prognostic tools and are less useful for geriatric patients with multi-morbidity or frail older persons without severe comorbidities.

Second, there are tools specifically for detection of patients with palliative care needs and to make the transition from cure to care. Four of those tools are well described in literature: the Gold Standards Framework Proactive Indicator Guidance (GSF PIG), the Supportive and Palliative Care Indicators Tool (SPICT), the PALliative necessities ccOMS-icO (necPAL), and the rADboud indicators for PAlliative care needs (rADPAc) [14]. They have not been validated specifically in older patients, except for SPICT [19].

Third, there are tools for older patients with chronic, non-oncological diseases including limitations in Activities of Daily Living (ADL), gait speed, hand grip strength or history of falls. These geriatric tools are mostly developed to predict functional decline and mortality within 3–10 years.

The clinical goal is to start palliative care timely and to better identify older hospitalized patients with high risk of dying within a short term time frame. Most of the geriatric tools are not validated for one-year mortality. Moreover, these studies mainly include healthy, community-dwelling older men and women [20–24]. An exception is the Multidimensional Prognostic Index (MPI), a tool based on the Comprehensive Geriatric Assessment (CGA) to predict mortality in older hospitalized patients [25].

Usual gait speed is a quick, safe and inexpensive instrument that is often put forward as a good prognostic factor in older persons. Usual gait speed has already been assessed in several studies and has shown an association with long-term mortality [26–28].

### Goals of research

In this prospective study, we will assess if usual gait speed can be used as a prognostic factor for early palliative care identification in older hospitalized patients. We will examine patients on two different wards, a geriatric ward and a disease specific ward. In general, patients on a geriatric ward are supposed to be more frail and have more comorbidities.

As the harms of a false negative result (deny patients palliative care) outweigh the harms of a false positive result (possibly starting palliative care principles too early) [29, 30], we value sensitivity more important than specificity. We aim at a sensitivity of more than 70%, a specificity of more than 50% and a AUC of ≥0.70. The choice of a cut-off is driven by these considerations.

The research questions are as follows:

1. Do the two different patient groups differ as hypothesized?
2. Is it feasible to perform usual gait speed in older persons admitted to the hospital on both wards?
3. What is the predictive accuracy and the optimal cut-off of usual gait speed for one-year mortality, aiming at a high sensitivity, on both wards?
4. Do patients who walk slower than the determined cut-off of usual gait speed also die earlier on both wards?

## Methods

### Design and setting

Patients were prospectively recruited from the hospital AZ Alma Eeklo, Belgium at the departments of cardiology and geriatrics. Acute geriatric wards usually perform comprehensive assessment and patient-centered care for frail and comorbid hospitalized older people, while the disease specific cardiology wards manage younger and older patients presenting with acute cardiovascular diseases without need for intensive care.

Patients of 75 years and older admitted on the ward for more than 2 days were eligible for inclusion. Patients who were younger than 75 years, who had been admitted on another ward for more than 48 h (such as coronary care unit, orthopedics or intensive care unit) before being admitted to geriatrics or cardiology, dying patients on admission or patients for whom this was not the first admission within the study period were excluded.

Data were obtained from standardized interviews and medical records.

### Ethics

The study was approved by the local ethics committee (Belgian Registration number B670201734355) and patients or legal representative of the incompetent patient were asked oral and written informed consent. We also asked approval for contacting them after 1 year.

### Data collection

From January to July 2018, data were collected prospectively and consecutively on three time points (admission, discharge and follow-up after 1 year). At time of admission, after informed consent, patient characteristics and quality of life was assessed. We also asked the patient his/her contact details and those of the legal representative. Data that are part of routine assessment of the patient were retrieved from the medical record at discharge: length of stay, survival, geriatric risk profile, comorbidities, nutritional status, cognition, functionality, type and seriousness of acute illness and usual gait speed. The usual gait speed measurement closest to discharge was used. After 1 year, survival status and time of death was assessed by telephone call. First contact was the patient, second the legal representative.

### Usual gait speed

Patients were asked to stand up and start walking at usual pace for 6 m. After two meters, the four meter walking time was measured. Patients could use their own walking aid. On the geriatric ward, usual gait speed was measured by the physiotherapist, on the cardiology ward by the occupational or physiotherapist of the geriatric liaison team. The most used cut-off value of usual gait speed in literature is 0.8 m/s [27].

The measurement of usual gait speed is standard clinical practice on the geriatric ward and for patients assessed by the geriatric liaison team. Since usual gait speed is our main variable, the patients without gait speed data were excluded in further analyses.

### Descriptive measures

Nutritional status was assessed using body mass index and the Nutritional Risk Screening (NRS) tool. NRS includes four questions and is an instrument to identify persons at risk for malnutrition (Additional file e-table 1) [31]. The Geriatric Risk Profile (GRP), a modified and translated version of the Triage Risk Screening Tool (TRST), was used to evaluate older persons for having a geriatric risk profile. The tool is mainly used on emergency departments and suggests if positive (cut-off score of 2) an increased risk of functional decline, hospitalizations and nursing home admissions (Additional file e-table 2) [32]. Functionality was assessed using the Katz index of ADL and the Lawton scale (instrumental Activities of Daily Living - iADL). Katz summarizes overall performance in bathing, dressing, going to the toilet, transferring, continence and feeding. Each item scores 1 (independent) to 4 (totally dependent) (Additional file e-table 3) [33]. Lawton iADL is an instrument to assess independent living skills in 8 domains. Each domain scores 0 (high dependency) or 1 (high independency) (Additional file e-table 4) [34]. Cognition was determined using the Mini Mental State Examination (MMSE). The maximum score is 30. A score of 23 or lower is indicative of cognitive impairment (Additional file e-table 5) [35]. Comorbidity was assessed by the Charlson Age-Comorbidity Index (CACI). This score is a combination of age and a measure of comorbidity to predict the relative risk of mortality. A higher score means a higher risk to die [36] (Additional file e-table 6).

### Statistical analysis

All analyses were performed with the SPSS statistical package version 25.

To determine whether the two different patient groups differ (research question 1) and to describe the number of missing values of usual gait speed and comparing characteristics of missing and no missing cases (research question 2), the Chi-Square test (for categorical) and the Mann Whitney U-test (for continuous variables) were used.

For research question 3, receiver-operator characteristic (ROC) curves and the area under the ROC curve (AUC) ± standard error were used. ROC curves and AUCs were developed from a logistic regression with usual gait speed as a single continuous predictor. Sensitivity, specificity and positive and negative predictive value were determined.

A cox model using dichotomized gait speed, which was additionally adjusted for age and sex was used for time to event analysis, to determine whether patients with a slow gait speed die earlier (research question 4). The test was not performed to estimate the exact moment of death. Outcomes were interpreted by Hazard Ratios (HR). The cox models were assessed for both wards. The binary aspect is of clinical importance since clinicians have to decide whether to start palliative care or not.

## Results

### Participants

**Fig. 1:**
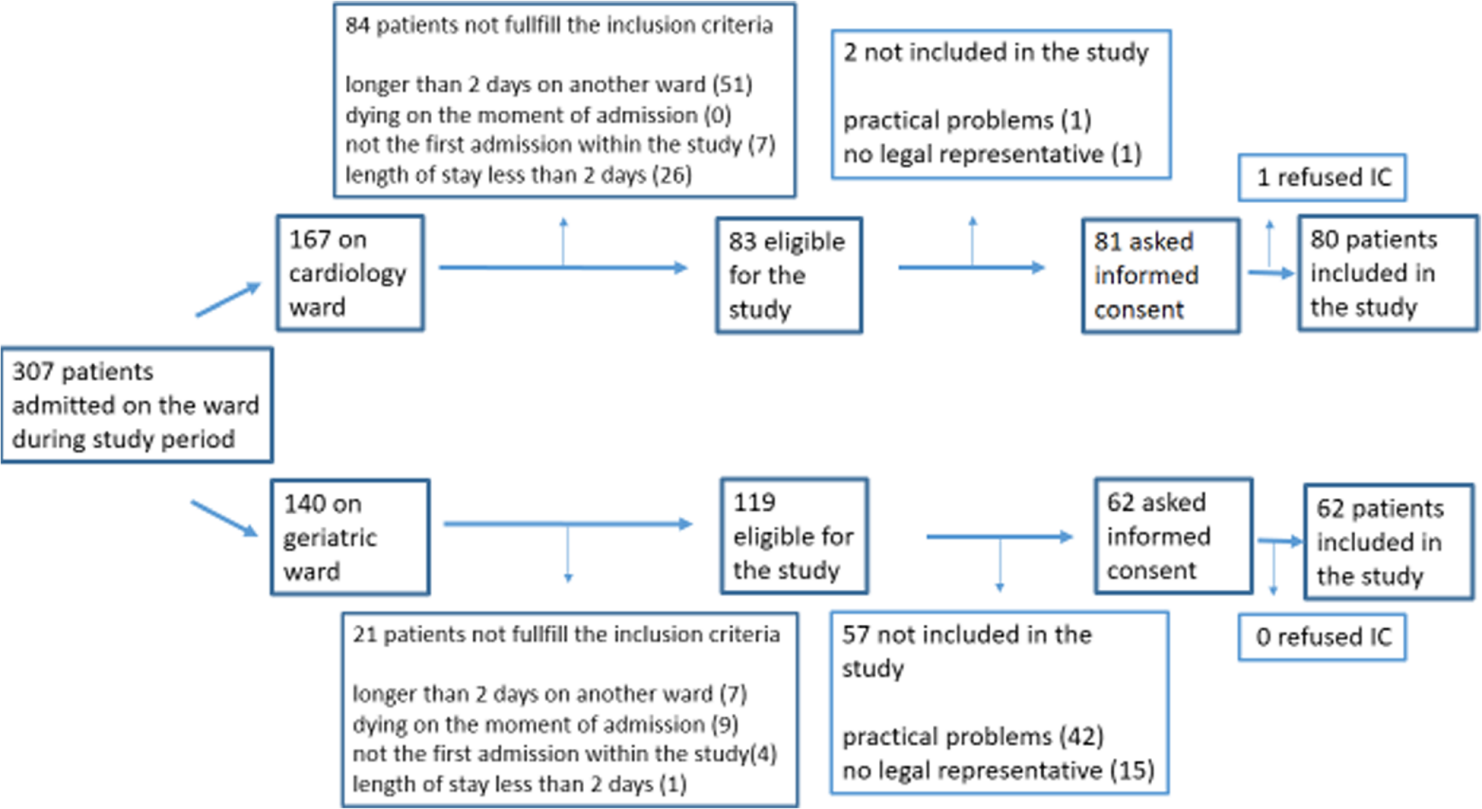
Flowchart of patients included in the study

On the geriatric ward, 119 patients were eligible. Fifty-seven patients were not included because of practical problems like absence of the researcher, too little time to include all patients admitted on the geriatric ward and absence of a legal representative. On cardiology, 83 patients were eligible and only two patients were not included (Fig. 1).

Characteristics of patients admitted on the acute geriatric and cardiology ward are shown in Table 1. Patients on the geriatric ward were older (86.6 years versus 82.5 years on cardiology), more often female, more frail as assessed by ADL and iADL and had lower nutritional status during the last months. Scores on cognition and comorbidities were not significantly different between the two wards.

**Table 1:** Basic descriptive statistics of the study participants expressed as number (%) or median (range)

			Acute geriatric ward *n* = 62 (MMSE *n* = 57)	Cardiology ward *n* = 80 (MMSE *n* = 10)	*p*-value
Age (years)	75–79		3 (4.8%)	23 (28.7%)	0.002
	80–84		19 (30.6%)	23 (28.7%)	
	85–89		19 (30.6%)	22 (27.5%)	
	90–94		17 (27.4%)	10 (12.5%)	
	95–100		4 (6.5%)	2 (2.5%)	
Sex	female		47 (75.8%)	37 (46.3%)	0.000
Residence	home		50 (80.6%)	76 (95.0%)	0.054
	short term stay in non-acute setting		5 (8.1%)	2 (2.5%)	
	nursing home		7 (11.3%)	2 (2.5%)	
Nutritional status	BMI (kg/m2)		25 (15–44)	25 (14–49)	0.021
	NRS total score		1 (0–4)	1 (0–4)	0.003
Frailty	GRP		4 (0–6)	2 (0–5)	0.467
	Functionality	Katz total score	13 (6–23)	7 (6–20)	0.002
		iADL (Lawton)	2 (0–7)	5 (0–7)	0.003
	Cognition	MMSE	22 (10–30)	23 (19–28)	0.124
Comorbidity	CACI		9 (4–15)	7 (3–15)	0.785
Length of stay in hospital			15.00 (1–91)	5.00 (1–34)	0.002
In-hospital mortality			1 (1.6%)	0 (0.0%)	0.254

*GRP* Geriatric Risk profile score, a modified and translated version of the triage risk screening tool (TRST), range 0–6, high score = high risk [32]; *Katz* evaluation scale for functional independence, range 6–24, high score = high dependency [33]; *iADL Lawton* instrumental Activities of Daily Living, range 0–7, high score = independence [34]; *NRS* Nutritional Risk Screening, range 0–4, high score = poor nutritional status [31]; *MMSE* Mini Mental State Examination, range 0–30, < 24/30 is an indicator of possible memory problems [35]; *CACI* Charlson Age-Comorbidity Index, a combination of age and a measure of comorbidity to predict the risk of mortality, high score = higher risk to die [36]

### Gait speed characteristics

Of the 142 participants (62 on the geriatric ward and 80 on the cardiology ward) included in the study, there were 18 missing values (13%) for usual gait speed, six (10%) on the acute geriatric ward and 12 (15%) on the cardiology ward. Comparison of characteristics of patients with and without a usual gait speed measurement showed significant difference for GRP (significantly lower geriatric risk profile in the group with usual gait speed missing) and iADL (significantly more independent on iADL in the group with usual gait speed missing). The other characteristics were not significantly different (Additional file e-table 7).

Mean usual gait speed on the acute geriatric ward and the cardiology ward was 0.33 m/s (95% CI 0.27–0.41) and 0.50 m/s (95% CI 0.43–0.59) respectively. On the geriatric ward, 51 out of 56 patients (91%) walked slower than 0.8 m/s; on the cardiology ward, 45 on 68 patients (66%) did.

### One-year outcome

The survival status of each patient could be ascertained. One-year mortality was not significantly different between acute geriatric (27%) and cardiology ward (15%) (*p* = 0.069).

**Fig. 2:**
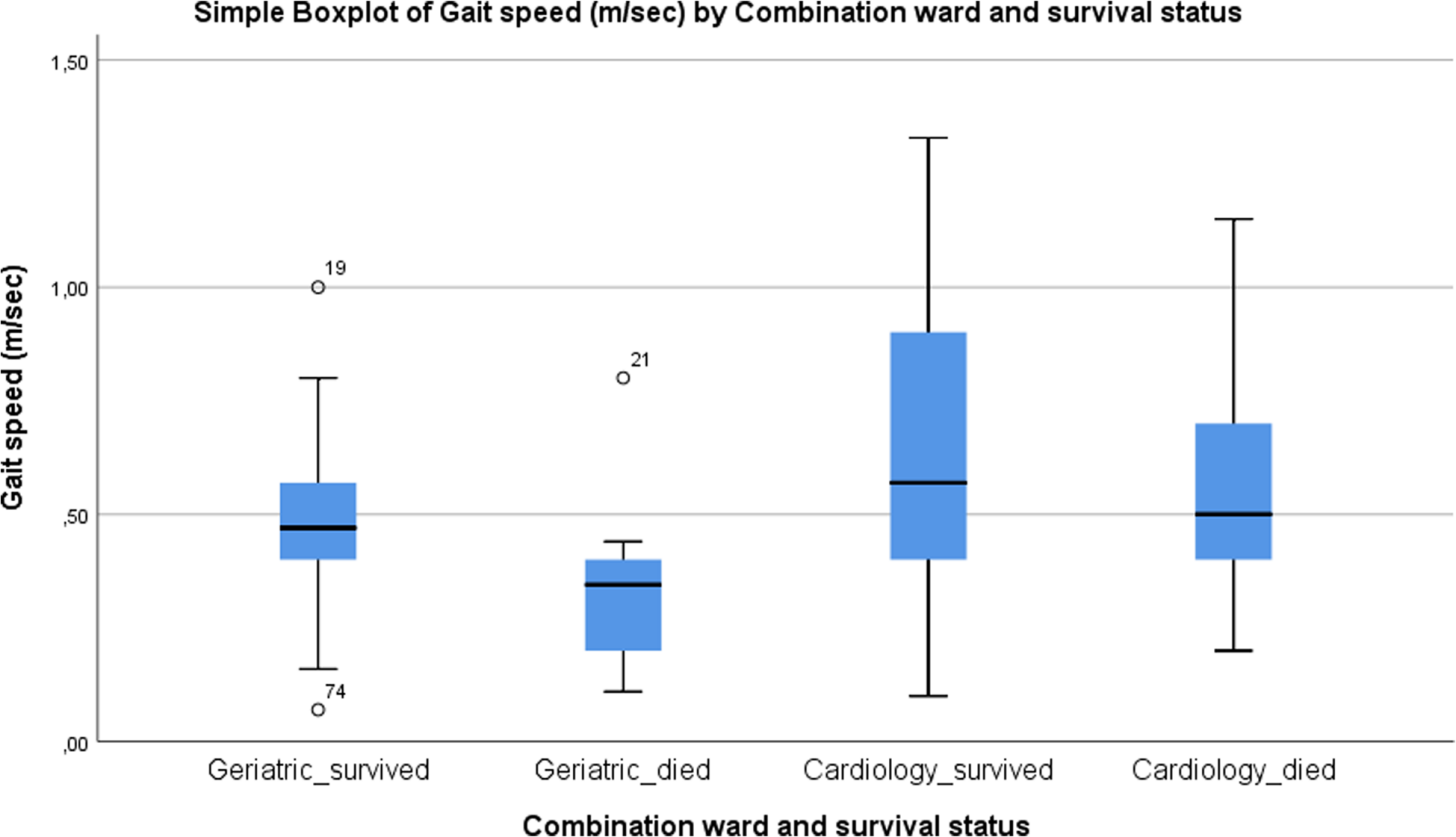
Usual gait speed for four groups of patients defined by ward and survival status

On the geriatric ward, mean usual gait speed for survivors was higher compared to non-survivors (respectively 0.36 m/s (95% CI 0.19–0.38) and 0.25 m/s (95% CI 0.29–0.47) (*p* = 0.005)) (Fig. 2). However, on the cardiology ward mean usual gait speed for survivors did not differ from non-survivors (respectively 0.51 m/s (95% CI 0.43–0.62) and 0.45 m/s (95% CI 0.31–0.81) (*p* = 0.559)) (Fig. 2).

### Diagnostic test accuracy

**Fig. 3:**
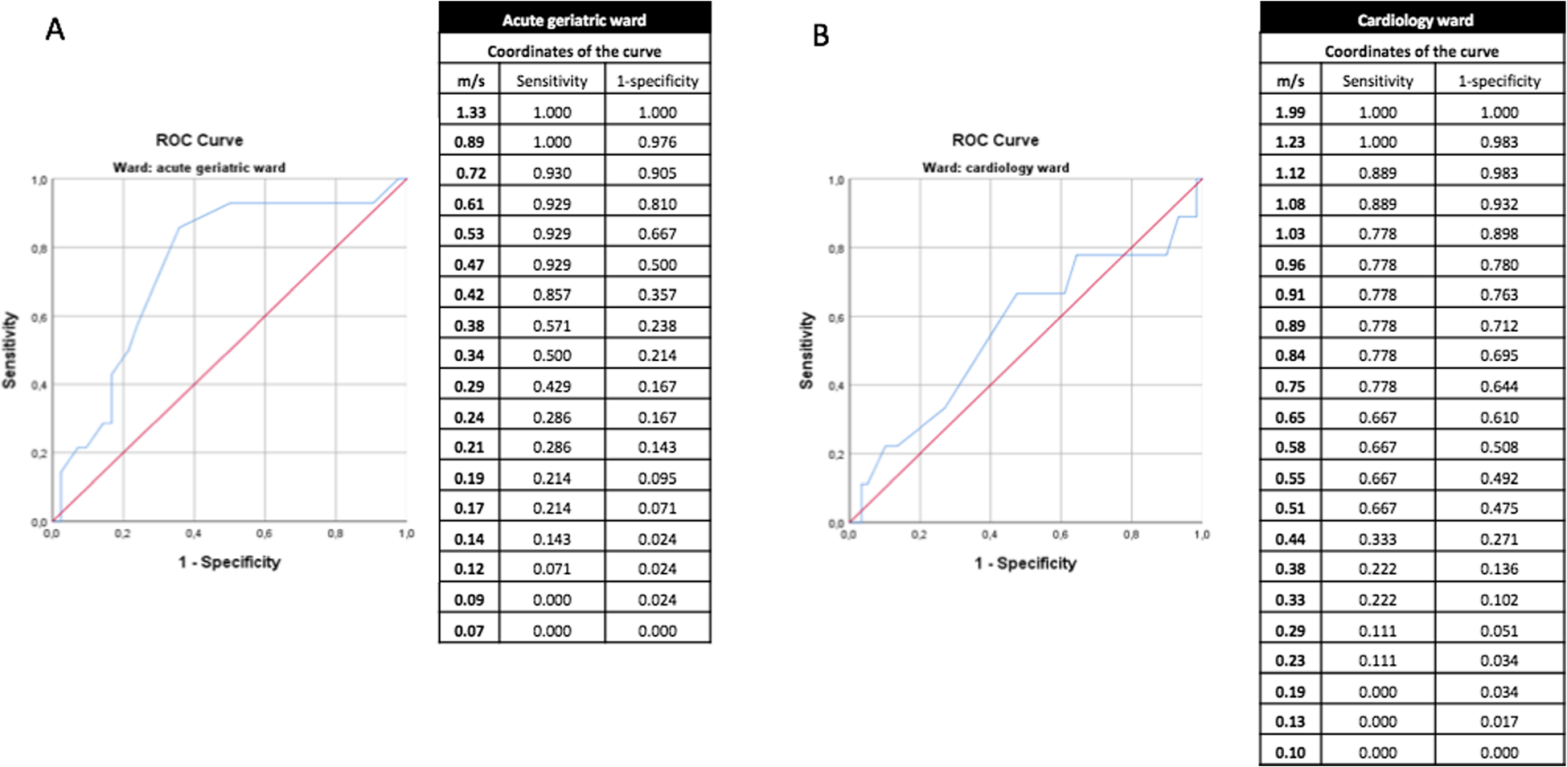
A and B. ROC curves of usual gait speed for the geriatric and cardiology ward

The ROC curve for usual gait speed on the geriatric ward revealed an AUC of 0.748 (*p* = 0.006). In this cohort, the best cut-off for one-year mortality was 0.42 m/s, with a sensitivity of 0.857 and a specificity of 0.643 (Fig. 3a). Twenty-seven patients (48.2%) hospitalized on the geriatric ward walked slower than 0.42 m/s. Twelve of them died within 1 year (44.4%). The positive and negative predictive value for one-year mortality are respectively 44.4 and 93.1%.

On the cardiology ward the AUC was 0.560 (*p* = 0.563) (Fig. 3b). The best possible cut-off for one-year mortality was 0.75 m/s, with a sensitivity of 0.778 and a specificity of 0.356. On the cardiology ward, 45 patients walked slower than 0.75 m/s (66.2%). Seven of them died within 1 year (15.6%). The positive and negative predictive value for one-year mortality are respectively 15.6 and 91.3%.

### Time to event analyses

**Fig. 4:**
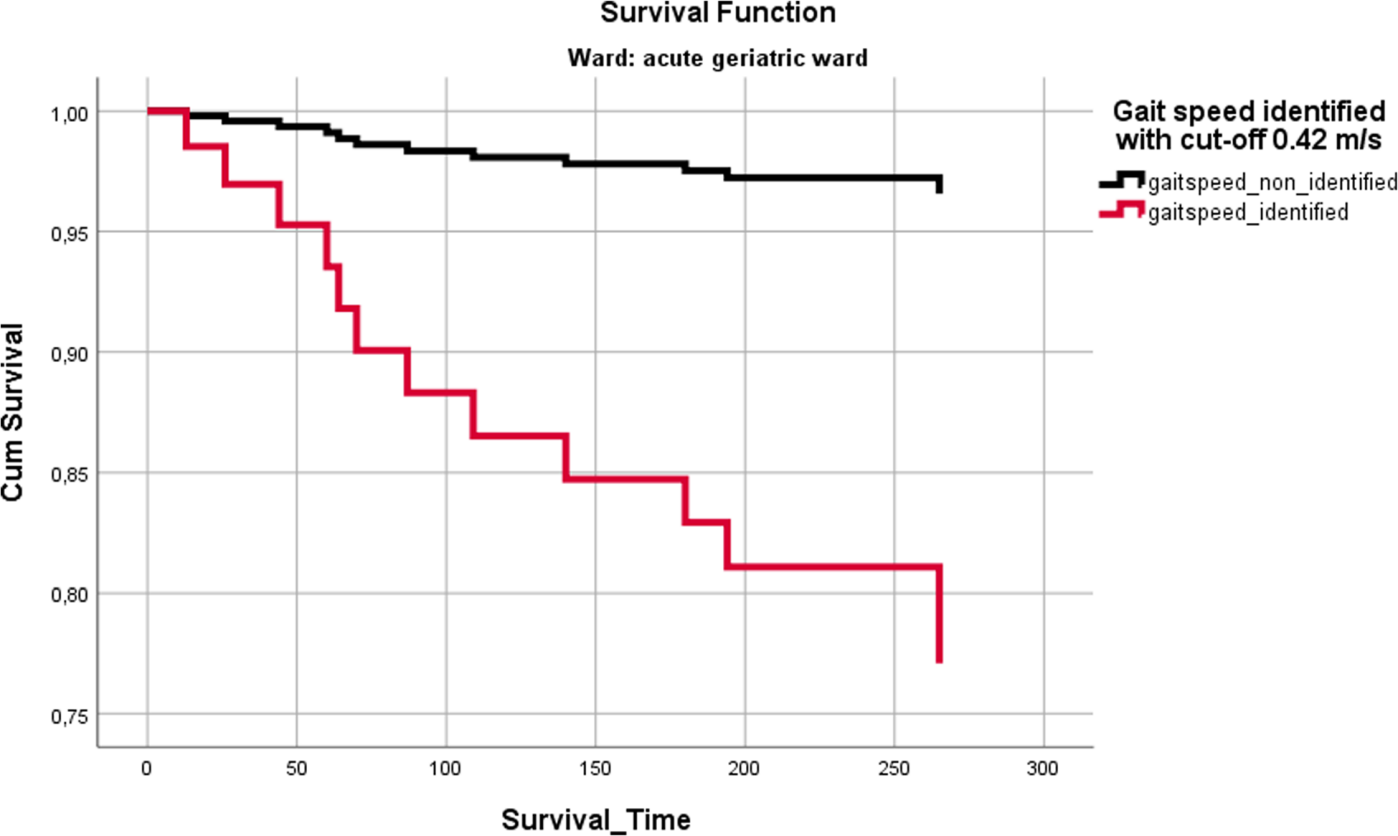
Survival curve calculated by COX regression after correction for age and sex

After correction for age and sex, usual gait speed showed a significant association with survival time using the 0.42 m/s cut-off on the geriatric ward (Fig. 4). Slow walkers died earlier than faster walkers (HR 7.456 (1.58–35.19); *p* = 0.011). On the cardiology ward, no significant association was shown for usual gait speed and survival time.

## Discussion

### Key results

In this prospective study, usual gait speed showed to be useful for early identification of palliative care for patients on the geriatric ward. The best cut-off value on the geriatric ward was lower than the cut-off of 0.8 m/s. Slow walkers died 7 times earlier.

### Interpretations

Our study puts usual gait speed forward as a prognostic factor for early palliative care identification in hospitalized older patients on a geriatric ward. Slow walkers also died 7 times earlier. However, the latter result does not influence the moment of starting palliative care. In older hospitalized persons with an expected life-time of one-year or less, palliative care should be introduced.

Usual gait speed has already been described in other studies to be a good prognostic factor, however mostly to assess long-term survival [26–28, 37, 38]. For example, in the analysis of Studenski et al. [26], describing a population of community-dwelling older persons, the AUC of usual gait speed was 0.7 to predict 5 and 10 year survival. The study of Hernandez-Luis et al. [38] described gait speed as a prognostic value for short term mortality, albeit in a younger population and also using the cut-off of 0.8 m/s. They showed that gait speed had a prognostic value on 100-day mortality (*p* = 0.005, HR 3.43) [38]. In the study of Afilalo et al., gait speed was studied for one-year mortality specifically following cardiac surgery [39].

In literature, we found some tools with comparable prognostic accuracy for one-year mortality in older persons. The CARING tool had an AUC of 0.86 [40]. Although, this was a retrospective study that included patients of ≥55 years who were admitted on the emergency unit and were subsequently hospitalized (on all wards, also the intensive care unit) [40]. In the prospective study of Ritt et al. [41] the Clinical Frailty Scale (CFS) had an AUC of 0.86 to predict one-year mortality in a population group of patients of ≥65 years who were hospitalized on geriatric wards. However, the latter tools are less feasible than usual gait speed, since a CGA is necessary to use the CFS and you need to go through the patient’s medical record for the CARING tool.

Usual gait speed is easy to perform and safe, with a good reproducibility [27, 37, 42–44]. In the study of Karpman et al. [45], usual gait speed was reported as a very acceptable measurement for both patients and clinical staff. Our study showed that usual gait speed as a part of standard clinical practice, is feasible in hospitalized patients since there were rather few missing values (18 out of 142). The absence of a usual gait speed measurement was due to a lack of time and other priorities during hospitalization. Patients included in the study without a usual gait speed measurement had a lower geriatric risk profile and were less dependent compared to those with a measurement. Thus, even in the frailer patients usual gait speed measurement was possible.

Contrary to literature in the field of usual gait speed, this study puts 0.42 m/s forward as the optimal cut-off value. The choice of the cut-off is based on clinical considerations by valuing sensitivity more important than specificity since starting palliative care principles too early is less harmful than denying patients palliative care. The most used cut-off value of usual gait speed is 1 m/s to predict long-term mortality and 0.8 m/s to predict adverse health outcomes [27]. These cut-offs proved not to be useful in this study with hospitalized older persons having a mean age of 86.6 years old, since only one patient had a gait speed above 0.8 m/s. In the analysis of Van Kan et al. [27], based on community-dwelling older persons, a cut-off of 0.6 m/s was proposed to identify the risk of further decline in already functionally impaired older adults. The best cut-off value in our study to predict mortality on the geriatric ward was even lower. This is similar to Ostir et al. [42] showing that a usual gait speed of less than 0.4 m/s had significantly longer lengths of stay and significantly decreased odds of home discharge. However, since the sample sizes in our study are small, the proposed cut-off value should be interpreted with caution. Larger studies are necessary to confirm the cut-off.

On the cardiology ward, the AUC was low (0.563). Several hypotheses can possibly explain the difference in prognostic accuracy between the two wards. First hypothesis is based on the observation that the type of patients and underlying morbidities are different between the two wards. On the geriatric ward, patients were more frail, expressed by the scores on ADL and iADL. We may assume that on the geriatric ward, patients mainly die because of complications of frailty. Usual gait speed is supposed to be more associated with frailty than with cardiovascular diseases. On the geriatric ward, multiple causes for frailty are combined: vascular, renal, respiratory, cognitive, together with sarcopenia and malnutrition. On the cardiology ward, people possibly more often have serious cardiovascular diseases leading to sudden cardiac death.

A second hypothesis is that the prognostic accuracy is underestimated in the cardiology ward because the sickest and fittest patients were excluded. Fifty-one patients were transferred from another ward (mostly the Coronary Care Unit), and thus excluded, compared to 7 on the geriatric ward while 26 had a length of stay of less than 48 h compared to one on the geriatric ward.

### Strengths and limitations

This study has several strengths. First of all, it is a prospective study with few missing values for usual gait speed, even in the frail older patients. Secondly, to the best of the authors’ knowledge, it is the first study assessing usual gait speed on two different wards in the old hospitalized patient (mean age of 84 years). Thirdly, patients were included with a wide range of health status and functionality.

However, some potential limitations need to be acknowledged. Our study has small sample sizes. Since the percentage of patients who died within one-year is rather low, the suggested cut-off values should be confirmed in larger studies. Furthermore, we lossed statistical power by the use of cut-off values, but the goal of this study is to help clinicians in the decision-making of starting palliative care. Therefore, a cut-off value is necessary.

The study is unicentric and selection bias cannot be excluded; (1) one fifth of the eligible patients were not included because of practical problems. We cannot exclude bias since we have no patient characteristics of those patients; (2) patients who stayed more than 2 days on another ward or patients hospitalized for less than 2 days, were excluded. Thereby, we probably excluded both the sickest and fittest older patients. Another limitation is the absence of a standardized method of usual gait speed measurement. This makes it difficult to compare with other studies. In our study, patients were asked to walk 6 m. After two meters, the four meter walking time was measured. It was measured the same each time. However, other studies for example do not allow the use of a walking aid or start the measurement from standing. The latter can make a difference, especially in patients with Parkinson disease or polyarthritis.

### Future research

Future research will be necessary for external validation of the study results. Also, the proposed cut-off value of 0.42 m/s should be further investigated as well as longitudinal assessments of gait speed and association with mortality.

## Conclusion

In conclusion, our study showed that usual gait speed has potential to be included in clinical practice as a prognostic factor for early palliative care identification in hospitalized older patients on a geriatric ward.

## Supplementary Information

**Additional file 1 :** E-Table 1. Nutritional risk screening (NRS).

**Additional file 2 :** E-Table 2. Geriatric Risk Profile (GRP) a modified and translated version of the Triage Risk Screening Tool (TRST).

**Additional file 3 :** E-Table 3. Katz Activities of Daily Living (ADL): Evaluation scale for functional independence.

**Additional file 4 :** E-Table 4. Lawton instrumental Activities of Daily Living (iADL).

**Additional file 5 :** E-Table 5. Mini Mental State Examination (MMSE).

**Additional file 6 :** E-Table 6. Charlson Age-Comorbidity Index.

**Additional file 7 :** E-Table 7. non-respons bias: Basic descriptive statistics of participants with and without gait speed expressed as number (%) and median (range).

## Data Availability

The datasets used and/or analysed during the current study are available from the corresponding author on reasonable request.

## Abbreviations

ROC: Reveiver operating characteristic
iADL: instrumental Acitivities of Daily Living
AUC: Area under curve
HR: Hazard ratio
WHO: World Health Organization
GSF PIG: the Gold standards framework proactive indicator guidance
SPICT: the supportive and palliative care indicators tool
necPAL: The PALliative necessities ccOMS-icO
RADPAc: the rADboud indicators for PALliative care needs
ADL: Activities of daily living
MPI: Multidimensional prognostic index
CGA: Comprehensive geriatric assessment
NRS: Nutritional risk screening
GRP: Geriatric risk profile
TRST: The Triage risk screening tool
MMSE: Mini mental state examination
CACI: Charlson age-comorbidity index
CI: Confidence interval
CFS: Clinical frailty scale
