# Supplementary material for: Can usual gait speed be used as a prognostic factor for early palliative care identification in hospitalized older patients? A prospective study on two different wards
Source: BMC Geriatr. 2020 Nov 24;20:499. doi: 10.1186/s12877-020-01898-w (PMC7687723; doi:10.1186/s12877-020-01898-w)
Supplement: Supplementary file 1 — Additional file 1 : E-Table 1. Nutritional risk screening (NRS). [file 12877_2020_1898_MOESM1_ESM.pdf]

Additional file 1 - NRS

**E-table 1: Nutritional risk screening (NRS) (**Kondrup J, Rasmussen HH, Hamberg O, Stanga Z, Ad Hoc EWG. Nutritional risk screening (NRS 2002): a new method based on an analysis of controlled clinical trials. Clin Nutr. 2003;22(3):321-36.)	
Is the patient's body-mass-index (BMI) less than 20.5? (BMI = weight/height m²)	☐ Yes = 1<br>☐ No = 0
Has the patient lost weight within the last three months?	☐ Yes = 1<br>☐ No = 0
Has the patient had a reduced dietary intake in the last week?	☐ Yes = 1<br>☐ No = 0
Is the patient severely ill (e.g. in intensive therapy)?	☐ Yes = 1<br>☐ No = 0
**NRS total score:**	______ / 4
