# Supplementary material for: Can usual gait speed be used as a prognostic factor for early palliative care identification in hospitalized older patients? A prospective study on two different wards
Source: BMC Geriatr. 2020 Nov 24;20:499. doi: 10.1186/s12877-020-01898-w (PMC7687723; doi:10.1186/s12877-020-01898-w)
Supplement: Supplementary file 2 — Additional file 2 : E-Table 2. Geriatric Risk Profile (GRP) a modified and translated version of the Triage Risk Screening Tool (TRST). [file 12877_2020_1898_MOESM2_ESM.pdf]

Additional file 2 - GRP

**E-table 2: Geriatric Risk Profile (GRP) a modified and translated version of the Triage Risk Screening Tool (TRST).** (Meldon SW, Mion LC, Palmer RM, Drew BL, Connor JT, Lewicki LJ, et al. A brief risk-stratification tool to predict repeat emergency department visits and hospitalizations in older patients discharged from the emergency department. Acad Emerg Med. 2003;10(3):224-32.)	
History of cognitive impairment	☐ Yes = 2<br>☐ No = 0
Lives alone and/or no available caregiver	☐ Yes = 1<br>☐ No = 0
Difficulty walking/transferring or recent falls	☐ Yes = 1<br>☐ No = 0
Hospitalization in previous 90 days	☐ Yes = 1<br>☐ No = 0
Five or more medications	☐ Yes = 1<br>☐ No = 0
**GRP total score**	**___ / 6**
