# Supplementary material for: Can usual gait speed be used as a prognostic factor for early palliative care identification in hospitalized older patients? A prospective study on two different wards
Source: BMC Geriatr. 2020 Nov 24;20:499. doi: 10.1186/s12877-020-01898-w (PMC7687723; doi:10.1186/s12877-020-01898-w)
Supplement: Supplementary file 3 — Additional file 3 : E-Table 3. Katz Activities of Daily Living (ADL): Evaluation scale for functional independence. [file 12877_2020_1898_MOESM3_ESM.pdf]

Additional file 3 – ADL

E-table 3: Katz Activities of Daily Living (ADL): Evaluation scale for functional independence (Katz S, Ford AB, Moskowitz RW, Jackson BA, Jaffe MW. Studies of Illness in the Aged. The Index of Adl: A Standardized Measure of Biological and Psychosocial Function. JAMA. 1963;185:914-9.)

**Bathing**	**Score**
☐ Requires no help with bathing	= 1
☐ Partial aid needed to wash upper or lower body	= 2
☐ Partial aid needed to wash upper and lower body	= 3
☐ Completely dependent for bathing	= 4
**Dressing**	
☐ Gets clothes from closets and drawers and puts on clothes and outer garments complete with fasteners	= 1
☐ Partial aid needed to dress upper or lower body (not taking in account shoelaces)	= 2
☐ Partial aid needed to dress upper and lower body	= 3
☐ Needs to be completely dressed	= 4
**Transferring**	
☐ Moves in and out of bed or chair unassisted	= 1
☐ Moves in and out of bed or chair independently with the use of mechanical transferring aides (wheelchair, crutches,…)	= 2
☐ Needs help from others in moving from bed to chair	= 3
☐ Bedridden or in a wheelchair and requires a complete transfer	= 4
**Toileting**	
☐ Goes to the toilet, gets on and off, arranges clothes, cleans genital area without help	= 1
☐ Needs help with one of the three items: goes to toilet, arranges clothes and cleans genital area	= 2
☐ Needs help with two of the three items: goes to toilet, arranges clothes and cleans genital area	= 3
☐ Needs help with the three items: goes to toilet, arranges clothes and cleans genital area	= 4
**Continence**	
☐ Exercises complete self-control over urination and defecation	= 1
☐ Accidental incontinent of bowel or bladder (incl. urinary catheter and artificial anus)	= 2
☐ Incontinent of bowel or bladder	= 3
☐ Incontinent of bowel and bladder	= 4
**Self-feeding**	
☐ Eating and drinking independently	= 1
☐ Needs help in preparing the meal or the drinks	= 2
☐ Partial help needed with feeding or drinking	= 3
☐ Completely dependent to eat or drink	= 4
	**Katz total score: ___ / 24**
