# Supplementary material for: Can usual gait speed be used as a prognostic factor for early palliative care identification in hospitalized older patients? A prospective study on two different wards
Source: BMC Geriatr. 2020 Nov 24;20:499. doi: 10.1186/s12877-020-01898-w (PMC7687723; doi:10.1186/s12877-020-01898-w)
Supplement: Supplementary file 4 — Additional file 4 : E-Table 4. Lawton instrumental Activities of Daily Living (iADL). [file 12877_2020_1898_MOESM4_ESM.pdf]

Additional file 4 - iADL

**E-table 4: Lawton instrumental Activities of Daily Living (iADL)** (Lawton MP, Brody EM. Assessment of older people: self-maintaining and instrumental activities of daily living. Gerontologist. 1969;9(3):179-86.)	
**Ability to use telephone**	**Score**
☐ Operates telephone on own initiative; looks up and dials numbers<br>☐ Dials a few well-known numbers<br>☐ Answers telephone, but does not dial<br>☐ Does not use telephone at all	= 1<br>= 1<br>= 1<br>= 0
**Shopping**	
☐ Takes care of all shopping needs independently<br>☐ Shops independently for small purchases<br>☐ Needs to be accompanied on any shopping trip<br>☐ Completely unable to shop	= 1<br>= 0<br>= 0<br>= 0
**Food Preparation**	
☐ Plans, prepares, and serves adequate meals independently<br>☐ Prepares adequate meals if supplied with ingredients<br>☐ Heats and serves prepared meals or prepares meals but does not maintain adequate diet<br>☐ Needs to have meals prepared and served	= 1<br>= 0<br>= 0<br><br>= 0
**Housekeeping**	
☐ Maintains house alone with occasion assistance (heavy work)<br>☐ Performs light daily tasks such as dishwashing, bed making<br>☐ Performs light daily tasks, but cannot maintain acceptable level of cleanliness Needs help with all home maintenance tasks<br>☐ Does not participate in any housekeeping tasks	= 1<br>= 1<br>= 1<br>= 1<br>= 0
**Mode of Transportation**	
☐ Travels independently on public transportation or drives own car<br>☐ Arranges own travel via taxi, but does not otherwise use public transportation<br>☐ Travels on public transportation when assisted or accompanied by another<br>☐ Travel limited to taxi or automobile with assistance of another<br>☐ Does not travel at all	= 1<br>= 1<br>= 1<br>= 0<br>= 0
**Responsibility for Own Medications**	
☐ Is responsible for taking medication in correct dosages at correct time<br>☐ Takes responsibility if medication is prepared in advance in separate dosages<br>☐ Is not capable of dispensing own medication	= 1<br>= 0<br>= 0
**Ability to Handle Finances**	
☐ Manages financial matters independently (budgets, writes checks, pays rent and bills, goes to bank); collects and keeps track of income<br>☐ Manages day-to-day purchases, but needs help with banking, major purchases, etc<br>☐ Incapable of handling money	= 1<br><br>= 1<br>= 0
**iADL total score: ___ / 7**	
