# Supplementary material for: Can usual gait speed be used as a prognostic factor for early palliative care identification in hospitalized older patients? A prospective study on two different wards
Source: BMC Geriatr. 2020 Nov 24;20:499. doi: 10.1186/s12877-020-01898-w (PMC7687723; doi:10.1186/s12877-020-01898-w)
Supplement: Supplementary file 5 — Additional file 5 : E-Table 5. Mini Mental State Examination (MMSE). [file 12877_2020_1898_MOESM5_ESM.pdf]

Additional file 5 - MMSE

**E-table 5: Mini Mental State Examination (MMSE)** Van den Beuken L, Huijskens, J., Nicolaes, L., & Van Engelen, E. Uitgebreide toelichting van het meetinstrument: Mini-Mental State Examination (MMSE). Geraadpleegd op 8 november, 2019, van https://meetinstrumentenzorgblobcorewindowsnet/test-documents/Instrument365/MMSEformpdf 2011.	
**Questions**	**Maximum score**
☐ Year?<br>☐ Season?<br>☐ Date?<br>☐ Day?<br>☐ Month?	5
☐ Where are we now?<br>☐ State?<br>☐ County?<br>☐ Town/city?<br>☐ Hospital?<br>☐ Floor?	5
The examiner names three unrelated objects clearly and slowly, then the instructor asks the patient to name all three of them.<br>The patient’s response is used for scoring. The examiner repeats them until patient learns all of them, if possible.	3
I would like you to count backward from 100 by sevens. (93, 86, 79, 72, 65, ...)<br>Alternative: Spell WORLD backwards.(D-L-R-O-W)	5
Earlier I told you the names of three things. Can you tell me what those were?	3
Show the patient two simple objects, such as a wristwatch and a pencil, and ask the patient to name them.	2
Repeat the phrase: ‘No ifs, ands, or buts.’	1
Take the paper in your right hand, fold it in half, and put it on the floor.<br>(The examiner gives the patient a piece of blank paper.)	3
Please read this and do what it says. (Written instruction is ‘Close your eyes.’)	1
Make up and write a sentence about anything.<br>(This sentence must contain a noun and a verb.)	1
Please copy this picture.<br>(The examiner gives the patient a blank piece of paper and asks him/her to draw the symbol below. All 10 angles must be present and two must intersect.)	1
	**Total score: /30**
