# Supplementary material for: Can usual gait speed be used as a prognostic factor for early palliative care identification in hospitalized older patients? A prospective study on two different wards
Source: BMC Geriatr. 2020 Nov 24;20:499. doi: 10.1186/s12877-020-01898-w (PMC7687723; doi:10.1186/s12877-020-01898-w)
Supplement: Supplementary file 6 — Additional file 6 : E-Table 6. Charlson Age-Comorbidity Index. [file 12877_2020_1898_MOESM6_ESM.pdf]

Additional file 6 – Charlson Age-Comorbidity Index

**E-table 6: Charlson Age-Comorbidity Index** (Charlson M, Szatrowski TP, Peterson J, Gold J. Validation of a combined comorbidity index. J Clin Epidemiol. 1994;47(11):1245-51.)	
Age (years)	Scores 0 point. Each decade of age ≥ 50 years is equivalent to a 1-point increase in comorbidity (ie, 50-59 years = 1 point, 60-69 years = 2point)
AIDS<br>Metastatic solid tumor	+ 6 points
Moderate or severe liver disease	+ 3 points
Any non-metastatic solid tumor<br>Malignant lymphoma<br>Leukemia<br>Diabetes with end organ damage<br>Moderate or severe renal disease<br>Hemiplegia	+ 2 points
Diabetes without end organ damage<br>Mild liver disease<br>Ulcer disease<br>Connective tissue disease<br>Chronic pulmonary disease<br>Dementia<br>Cerebrovascular disease<br>Peripheral vascular disease<br>Congestive heart failure<br>Myocardial infarction	+ 1 point
