# Supplementary material for: Can usual gait speed be used as a prognostic factor for early palliative care identification in hospitalized older patients? A prospective study on two different wards
Source: BMC Geriatr. 2020 Nov 24;20:499. doi: 10.1186/s12877-020-01898-w (PMC7687723; doi:10.1186/s12877-020-01898-w)
Supplement: Supplementary file 7 — Additional file 7 : E-Table 7. non-respons bias: Basic descriptive statistics of participants with and without gait speed expressed as number (%) and median (range). [file 12877_2020_1898_MOESM7_ESM.pdf]

Additional file 7 – Non-respons bias

E-table 7: non-respons bias: Basic descriptive statistics of participants with and without gait speed expressed as number (%) and median (range).

			Gait speed measurement available n = 124 (MMSE n = 61)	Missings for gait speed measurement n = 18 (MMSE n = 6)	p-value
Age (years)	75-79		21 (16.9%)	5 (27.8%)	0.271
	80-84		36 (29.0%)	6 (33.3%)	
	85-89		38 (30.6%)	3 (16.7%)	
	90-94		25 (20.2%)	2 (11.1%)	
	95-100		4 (3.2%)	2 (11.1%)	
Sex	female		72 (58.1%)	12 (66.7%)	0.488
Residence	Home		109 (87.9%)	17 (94.4%)	0.774
	Short term stay in non-acute setting		7 (5.6%)	0 (0.0%)	
	Nursing home		8 (6.5%)	1 (5.6%)	
Nutritional status	BMI (kg/m2)		25 (15-44)	24 (14-49)	0.880
	NRS total score		1 (0-4)	1 (0-4)	0.079
Frailty	GRP		3 (0-6)	2 (0-5)	0.007
	Functionality	Katz total score	9 (6-23)	7.5 (6-20)	0.418
		iADL Lawton	3 (0-7)	5 (0-7)	0.022
	Cognition	MMSE	22 (10-30)	24 (19-30)	0.220
Comorbidity	CACI		8 (3-15)	7 (3-12)	0.152
Length of stay in hospital			9.00 (1-91)	6.50 (2-30)	0.360

GRP = Geriatric Risk profile score, a modified and translated version of the triage risk screening tool (TRST), range 0-6, high score = high risk (1); Katz = evaluation scale for functional independence, range 6-24, high score = high dependency (2); iADL Lawton = instrumental Activities of Daily Living, range 0-7, high score = independency (3); NRS = Nutritional Risk Screening, range 0-4, high score = poor nutritional status (4); MMSE = Mini Mental State Examination, range 0-30, <24/30 is an indicator of possible memory problems (5); CACI = Charlson Age-Comorbidity Index, a combination of age and a measure of comorbidity to predict the risk of mortality, high score = higher risk to die (6).

(1) Meldon SW, Mion LC, Palmer RM, Drew BL, Connor JT, Lewicki LJ, et al. A brief risk-stratification tool to predict repeat emergency department visits and hospitalizations in older patients discharged from the emergency department. Acad Emerg Med. 2003;10(3):224-32.

(2) Katz S, Ford AB, Moskowitz RW, Jackson BA, Jaffe MW. Studies of Illness in the Aged. The Index of Adl: A Standardized Measure of Biological and Psychosocial Function. JAMA. 1963;185:914-9.

(3) Lawton MP, Brody EM. Assessment of older people: self-maintaining and instrumental activities of daily living. Gerontologist. 1969;9(3):179-86.

(4) Kondrup J, Rasmussen HH, Hamberg O, Stanga Z, Ad Hoc EWG. Nutritional risk screening (NRS 2002): a new method based on an analysis of controlled clinical trials. Clin Nutr. 2003;22(3):321-36.
(5) Van den Beuken L, Huijskens, J., Nicolaes, L., & Van Engelen, E. Uitgebreide toelichting van het meetinstrument: Mini-Mental State Examination (MMSE). . Geraadpleegd op 8 november, 2019, van https://meetinstrumentenzorgblobcorewindowsnet/test-documents/Instrument365/MMSEformpdf 2011.
(6) Charlson M, Szatrowski TP, Peterson J, Gold J. Validation of a combined comorbidity index. J Clin Epidemiol. 1994;47(11):1245-51.
